# Nanoenzyme-Enhanced Catalytic Photodynamic Therapy for Cancer

**DOI:** 10.3390/pharmaceutics18101291

**Published:** 2026-10-12

**Authors:** Mpho Mohlongo, Blassan P. George, Heidi Abrahamse

**Affiliations:** Laser Research Centre, Faculty of Health Sciences, University of Johannesburg, P.O. Box 1711, Doornfontein 2028, South Africa; 217039520@students.uj.ac.za (M.M.); blassang@uj.ac.za (B.P.G.)

**Keywords:** catalytic photodynamic therapy (CAT-PDT), nanoenzymes, tumour microenvironment (TME), tumour hypoxia, reactive oxygen species (ROS)

## Abstract

Catalytic photodynamic therapy is an emerging strategy that integrates enzyme-mimetic nanomaterials with photosensitisers to overcome the intrinsic limitations of conventional photodynamic therapy in solid tumours. Conventional photodynamic therapy relies on the oxygen-dependent generation of cytotoxic reactive oxygen species, which is severely compromised by tumour hypoxia, elevated antioxidant defences, heterogenous photosensitiser accumulation, and limited light penetration. Nanoenzyme-mediated catalytic photodynamic therapy addresses these barriers through two mechanistically distinct catalytic modes: oxygen-generating catalytic photodynamic therapy, in which catalase-like nanoenzymes disproportionate endogenous hydrogen peroxide into molecular oxygen to sustain Type II photochemistry, and oxygen-independent catalytic photodynamic therapy, in which peroxidase/Fenton-like nanoenzymes convert hydrogen peroxide into hydroxyl radicals without requiring oxygen or light activation. Multi-enzyme cascade systems integrate both modes, enabling synergistic reactive oxygen species generation across heterogenous tumour regions. This review discusses the catalytic mechanisms, nanoenzyme classes, including carbon-based materials, metal–organic frameworks, plasmonic nanostructures, and biomimetic systems, and therapeutic applications of catalytic photodynamic therapy. We critically evaluate the evidence linking catalytic activity to improved photodynamic outcomes, highlight methodological limitations in reactive oxygen species assessment, and examine translational challenges including substrate heterogeneity, colloidal scalability, uncontrolled reactive oxygen species generation, and regulatory hurdles. Emerging research directions, including artificial intelligence-mediated nanomaterial design, quantum-guided catalytic optimisation, and immunoengineering, are considered as strategies to accelerate clinical translation.

## 1. Introduction

Cancer is a leading cause of morbidity and mortality worldwide, accounting for millions of deaths annually [1,2]. Although conventional therapies, including surgery, chemotherapy, hormone therapy, and radiotherapy, remain the primary treatment modalities, their clinical effectiveness is frequently limited by systemic toxicity, tumour recurrence, and resistance mechanisms [3]. These challenges highlight the need for alternative strategies that improve therapeutic efficacy while maintaining minimal invasiveness. Thus, photodynamic therapy (PDT) has emerged as a promising therapeutic approach [4,5].

The principle of PDT is based on the interaction between three essential components: a photosensitiser (PS) molecule, light of an appropriate wavelength, and molecular oxygen. Following light activation, the PS transfers energy to surrounding oxygen molecules, producing cytotoxic reactive oxygen species (ROS). These ROS, such as singlet oxygen, induce oxidative damage to cellular membranes, proteins, nucleic acids, and other biomolecules, leading to cancer cell death. In contrast to conventional cancer therapies, PDT offers several advantages, including high selectivity, reduced systemic toxicity, minimal resistance development, and the ability to preserve surrounding healthy tissue. Consequently, PDT has been applied in the treatment of several superficial malignancies, including oesophageal, bladder, and non-melanoma skin cancers [4,5,6].

Despite these advantages, the effectiveness of PDT is limited in the treatment of solid tumours. This limitation arises primarily from the complex tumour microenvironment (TME), which directly interferes with the photochemical processes required for effective ROS generation. Hypoxia, resulting from abnormal tumour vasculature, poor perfusion, and high metabolic demand, limit oxygen availability, suppressing ROS production [7,8]. Additional barriers, including heterogeneous PS accumulation, elevated interstitial fluid pressure, dense extracellular matrix deposition, and restricted light penetration into deep tissues, further reduce treatment efficiency [9,10,11]. These biological and physical barriers explain why conventional PDT often produces inconsistent therapeutic responses in solid tumours [5,7,8].

Nanotechnology provides strategies to address these limitations by improving PS delivery, enhancing tumour targeting, and improving efficacy. Among these developments, nanoenzymes, which function as active therapeutic components, have emerged as a promising solution. Unlike conventional nanocarriers, nanoenzymes have intrinsic enzyme-mimetic catalytic activity, promoting their involvement in biochemical reactions within the TME [12,13]. These nanoenzymes, including catalases, peroxidases, oxidase, and superoxide dismutase, generate oxygen, amplify ROS production, deplete antioxidants, and modulate the biochemical conditions of the TME, enhancing the efficacy of PDT. Their catalytic activity is derived from structural characteristics, such as exposed metal centres, surface charge distribution, crystal defects, heteroatom doping, charge distribution, and quantum. Compared with natural enzymes, nanoenzymes generally exhibit greater stability under changes in pH and temperature conditions, resistance to denaturation, and improved tunability, making them suitable for biomedical applications [12,13,14].

The integration of nanoenzymes with PDT has led to the development of catalytic photodynamic therapy (CAT-PDT), an emerging therapeutic strategy designed to overcome the intrinsic limitations of conventional PDT [15]. In CAT-PDT, nanoenzymes actively regulate the TME by alleviating hypoxia, suppressing intracellular antioxidant defences, and sustaining ROS production. These catalytic nanosystems modulate biochemical processes that enhance photodynamic efficiency and therapeutic outcomes [16,17,18].

Carbon-based nanocatalysts, such as graphene derivatives and carbon dots, exhibit peroxidase-like activity and high photostability [19]. Nanoenzymes can also enhance PDT through photonic and electronic mechanisms: plasmonic nanoparticles (Au, Ag, Pd) improve light absorption through localised surface plasmon resonance (LSPR), carbon-based nanoenzymes facilitate charge separation and energy transfer, and metal–organic framework (MOF) nanoenzymes combine PS encapsulation with catalytic modulation in a single molecular structure [20,21,22]. Some nanoenzymes also catalyse the oxidation of glutathione, reducing antioxidant defences in tumour cells and sustaining ROS accumulation during PDT [21,23].

Artificial intelligence is increasingly being used to accelerate nanomaterial discovery and optimise catalyst design, while quantum-guided computational approaches are improving the prediction of catalytic behaviour. Together with developments in immunoengineering and multimodal therapy, these technologies are expected to facilitate the development of more effective and clinically translatable catalytic nanoplatforms [24].

**Figure 1:**
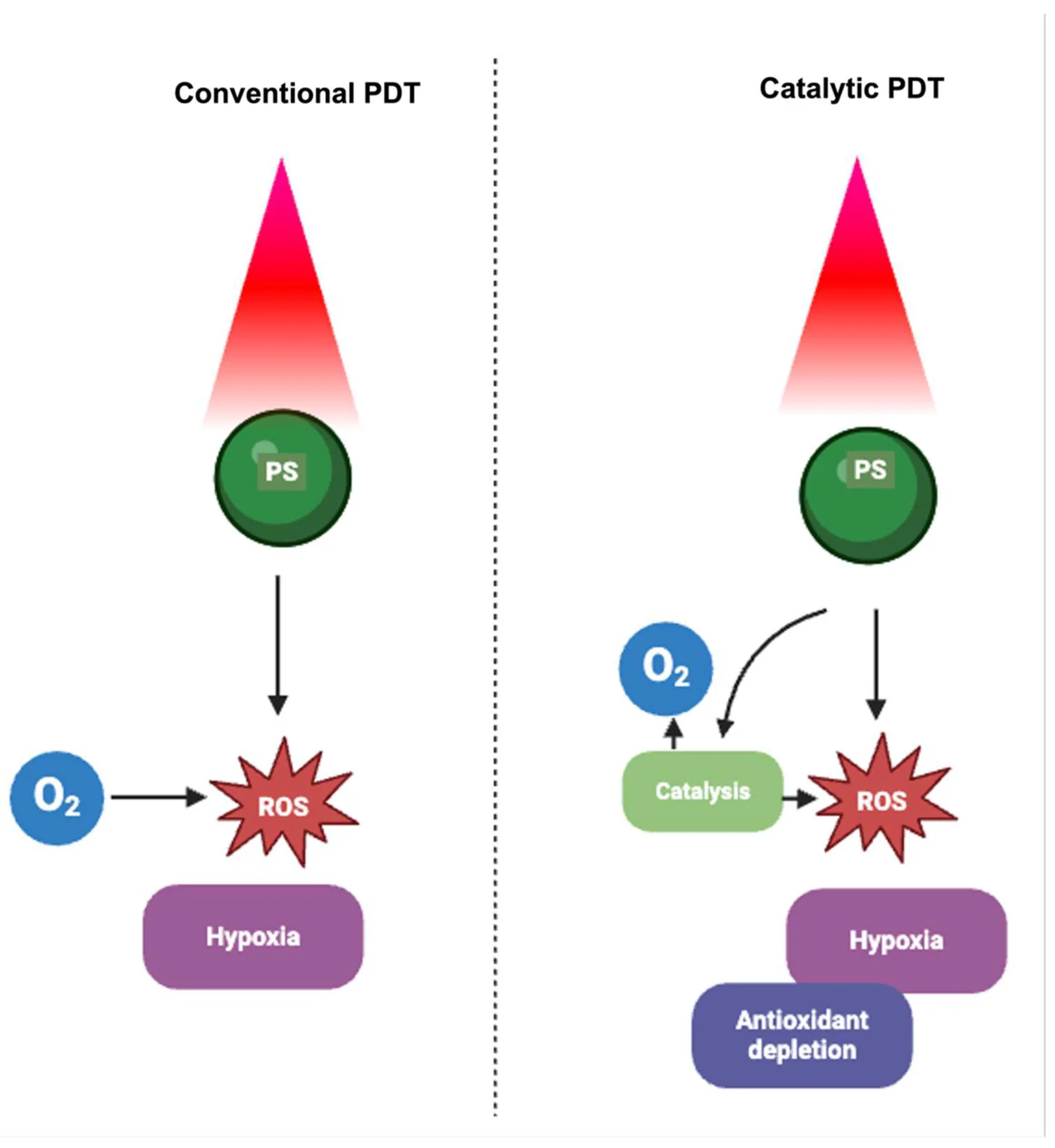
Comparison between conventional photodynamic therapy and nanoenzyme-enhanced catalytic photodynamic therapy. Conventional PDT relies on oxygen-dependent ROS generation following PS activation, which limits therapeutic efficacy under tumour hypoxia. CAT-PDT incorporates nanoenzyme-driven catalytic processes that regenerate oxygen and disrupt antioxidant buffering, resulting in sustaining ROS production and active modulation of the TME. (Created in BioRender.com. https://BioRender.com/v1aaltn).

In conventional PDT, light-activated PS molecules generate ROS in an oxygen-dependent manner, resulting in diminished efficacy under hypoxic tumour conditions [25]. In contrast, CAT-PDT incorporates nanoenzyme-mediated catalysis to actively regenerate oxygen, amplify ROS production, and deplete antioxidant defences within the TME [26]. These mechanisms are distinguished in Figure 1.

This review examines the design principles, CAT- mechanisms, material characteristic, biological interactions, therapeutic applications, and translational challenges of nanoenzyme-assisted PDT. It discusses the major classes of nanoenzymes, including metal-based, noble metal, carbon-based, MOF-derived and biomimetic platforms, and highlights emerging trends such as NIR-II and X-ray activated nanoenzymes, tumour-logic catalytic systems, and self-evolving catalytic materials. These advances highlight the potential of nanoenzymes to improve the precision, efficacy, and clinical translation of PDT.

Several recent reviews have examined nanoenzymes in cancer therapy, TME modulation, and nanodynamic therapies, including PDT. These studies have provided important overviews of nanoenzyme classes, catalytic mechanisms, tumour-responsive design, and combinations with different therapeutic modalities. However, the available literature has generally approached nanoenzyme-enabled PDT as one component of the broader nanodynamic therapy landscape or has emphasised material design and therapeutic applications rather than critically examining whether catalytic activity translates into PDT efficacy.

Therefore, this review adopts a more focused catalysis-to-therapy perspective. Rather than classifying nanoenzymes solely according to their material composition, we examine CAT-PDT according to the catalytic function introduced into the photodynamic system and the biological limitation that this catalytic activity is intended to overcome. Additionally, this review also evaluates dependence of catalytic activity on tumour-associated substrates, pH, oxygen availability, redox state and catalyst surface properties and the extent to which parameters are maintained when transitioning from simplified in vitro systems to heterogenous tumour environments.

Importantly, this review critically evaluates the evidence linking catalytic activity, changes in oxygen or ROS dynamics, enhanced PDT effects, and therapeutic efficacy in vivo. This approach highlights an important distinction between demonstrating enzyme-like catalytic activity experimentally and demonstrating that such activity is responsible for improved therapeutic outcomes. We also examine methodological limitations in ROS assessment, differences between in vitro and in vivo models, substrate availability, catalytic selectivity, nanoenzyme stability and reproducibility, and the pharmaceutical and regulatory barriers that must be addressed before CAT-PDT can progress toward clinical evaluation.

Accordingly, the principal contribution of this review is not simply to catalogue nano-enzyme-based PDT systems but to establish a critical framework for evaluating the mechanistic validity, experimental evidence, limitations, and translational readiness of nanoenzyme-enabled CAT-PDT.

### Literature Search Strategy

This review was conducted as a structured narrative review of the literature on nano-enzyme-enabled CAT-PDT for cancer. PubMed was searched using combinations of terms relating to nanoenzymes, photodynamic therapy, cancer, and specific enzyme-like CAT-PDT activities.

The search terms included combinations of “nanozyme”, “nanoenzyme”, “enzyme-mimetic nanomaterial”, “nanoenzyme-mediated catalytic photodynamic therapy”, “catalytic photodynamic therapy” “CAT-PDT”, “photodynamic therapy”, “PDT’, and “cancer”, with appropriate database-specific adaptations. The search covered publications from 2018 to September 2026, with the final search conducted in October 2026. Studies were considered relevant when they investigated nanoenzyme-based systems in which enzyme-like catalytic activity was used to enhance, modulate, or otherwise contribute to PDT for cancer treatment. Studies unrelated to cancer or PDT, studies focusing exclusively on other therapeutic modalities without a PDT component, and publications that did not address nanoenzyme-mediated catalytic mechanisms were excluded. Relevant review articles were additionally examined to identify seminal studies and recent developments.

For the historical development of CAT-PDT (Section 3.1), a targeted search was performed to identify seminal papers that represent key milestones in the field. Search terms included combinations of “nanozyme”, “nanoenzyme”, “enzyme-mimetic nanomaterial”, “nanoenzyme-mediated catalytic photodynamic therapy”, “catalytic photodynamic therapy” “CAT-PDT”, “photodynamic therapy”, “PDT’, “cancer”, AND “history”, “development”, and “milestone”. This search covered publications from 1990 to September 2026, with the final search conducted in October 2026.

This review is intended as a structured narrative review rather than a systemic review or scoping review. Therefore, no formal meta-analysis was performed, and studies were not quantitatively pooled.

## 2. Photodynamic Therapy and the Tumour Microenvironment

### 2.1. Overview of Photodynamic Therapy

PDT is a cancer treatment modality that offers spatial and temporal control, minimal invasiveness, and the potential for selective tumour cell destruction. The therapeutic mechanism involves three essential components: a PS, light of an appropriate wavelength, and molecular oxygen. Upon light irradiation, the PS transitions to an excited triplet state, transferring energy to molecules to generate singlet oxygen and other ROS. These ROS induce oxidative stress, damage cellular membranes, proteins, and nucleic acids, ultimately leading to cancer cell death by apoptosis, necrosis, or autophagy [27,28].

Clinically, PDT has demonstrated efficacy in dermatological tumours, head-and-neck cancers, and localised superficial malignancies. Its advantages include repeatability, spatiotemporal control, and compatibility with multimodal regimens. However, despite decades of development, PDT remains underutilised in deep-seated solid tumours [27,29].

### 2.2. Classical Mechanism of PDT and ROS Pathways

**Figure 2:**
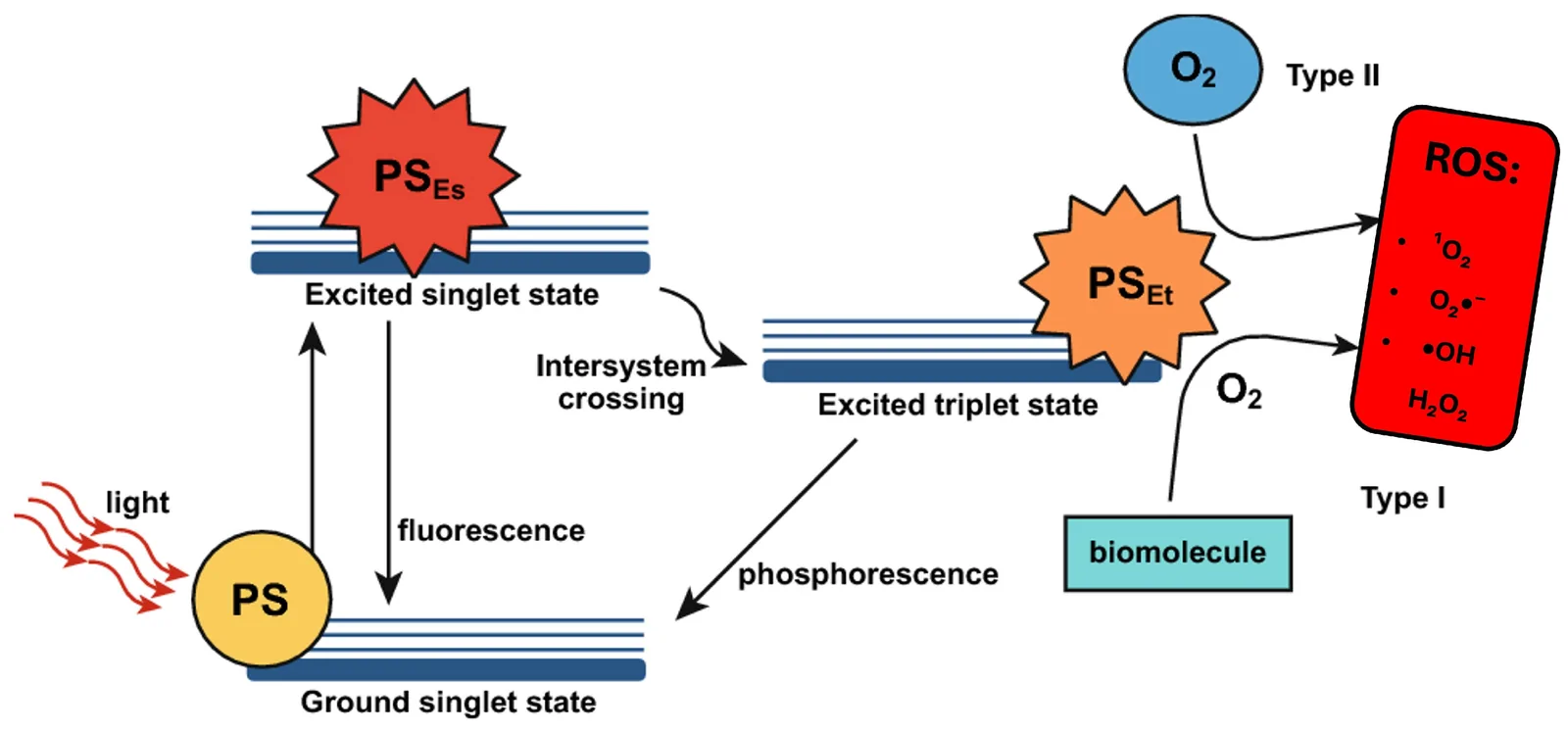
Classical photodynamic therapy mechanism and reactive oxygen species pathways. Following light absorption, the photosensitiser is excited to the singlet state (PS_Es). After intersystem crossing, the photosensitiser reaches the excited triplet state (PS_Et). From this common branch point, two competing parallel photochemical pathways occur. Type I reactions involve electron transfer to biomolecules and O_2_, generating O_2_•^−^, •OH, and H_2_O_2_. Type II reactions involve direct energy transfer from the excited triplet-state photosensitiser to ground-state O_2_, producing singlet oxygen (^1^O_2_). Both ^1^O_2_ and the Type I products are ROS that induce oxidative damage, leading to cell death. Type I and Type II are not sequential; the balance between them depends on photosensitiser structure, oxygen availability, and substrate concentration. Adapted from [33] under CC BY 4.0 (https://creativecommons.org/licenses/by/4.0/). Changes were made to group ^1^O_2_ with other ROS and to clarify the parallel nature of the pathways.

Following light absorption and intersystem crossing, the PS reaches an excited triplet state from which two competing photochemical pathways can occur: Type I and Type II reactions (Figure 2). In Type II pathways, the triplet PS transfers energy directly to ground-state molecular oxygen, producing singlet oxygen. Singlet oxygen is highly reactive and short-lived, yet it is the dominant cytotoxic agent in classical PDT. It reacts with unsaturated lipids, proteins, and nucleic acids, initiating oxidative damage that compromises membrane integrity, enzyme function, and genetic stability. In the Type I pathways, the triplet PS undergoes electron or hydrogen transfer reactions with nearby biomolecules, generating free radicals such as superoxide anion, hydroxyl radical, and hydrogen peroxide (H_2_O_2_); these secondary ROS amplify oxidative stress and contribute to cell death [30,31,32,33].

The relative contributions of the Type I and Type II pathways depend on the PS structure, oxygen availability, and the biochemical composition of the TME. Type II reactions require molecular oxygen for singlet oxygen generation, whereas Type I reactions can involve electron or hydrogen transfer to nearby substrates and may therefore be less sensitive to oxygen depletion at the initial photochemical step. However, downstream ROS formation in Type I reactions can also involve molecular oxygen. The balance between the two pathways therefore varies according to the physiochemical properties of the PS and the local cellular environment and TME [30,31,32].

ROS generated during PDT exert their cytotoxic effects through multiple mechanisms. At the cellular level, oxidative damage to mitochondrial membranes can promote release of cytochrome C and activation of caspase cascades, contributing to apoptosis. Severe oxidative stress can overwhelm repair systems, causing necrosis characterised by membrane rupture and uncontrolled cell lysis. PDT also damages tumour vasculature, inducing vascular shutdown and secondary hypoxia that deprives cancer cells of nutrients. Furthermore, PDT can stimulate immunogenic cell death, releasing danger-associated molecular patterns (DAMPs) that activate dendritic cells and prime anti-tumour immune responses. The biological outcomes of PDT are strongly dependent on the nature of the generated ROS, as each ROS exhibits distinct reactivity, diffusion capacity, and cytotoxic potential. The major ROS produced during PDT, together with their primary cellular targets, biological outcomes, and functional roles, are summarised in Table 1 [30,31,32].

Despite these powerful mechanisms, the efficiency of ROS generation is highly dependent on the TME. Hypoxia restricts the availability of oxygen for the Type II reactions, while elevated GSH and antioxidant enzymes neutralise ROS before they can exert cytotoxic effects. Dense extracellular matrices hinder the diffusion of both PS and oxygen, reducing the uniformity of ROS distribution. Light attenuation in deep tissues further limits the activation of PS molecules, confining ROS generation to superficial regions [34,35]. These factors collectively explain why PDT, although mechanistically robust, often fails to achieve durable responses in aggressive solid tumours. While both Type I and Type II pathways operate in conventional PDT, their efficiency is strongly constrained by oxygen availability and intracellular antioxidant defences, underscoring the need for catalytic strategies capable of sustaining ROS generation within hostile TME [34,35,36].

### 2.3. The Tumour Microenvironment as a Barrier to PDT

Despite the effectiveness of PDT, its use in deep-seated and solid tumours remains a challenge. The TME is a complex structure of cancer cells, stromal cells, immune cells, vasculature, extracellular matrix (ECM), and soluble factors. In addition to passively hosting tumour growth, the TME actively limits therapeutic responses. For PDT, the abnormal features of the TME counteract ROS generation and limit efficacy [27,37]. Hypoxia is one of the most significant barriers, as aberrant vasculature and impaired perfusion reduce oxygen availability, restricting the substrate required for ROS generation [27,38].

Elevated levels of GSH and antioxidant enzymes neutralize ROS, diminishing cytotoxicity and contributing to therapeutic resistance [39,40]. The ECM in solid tumours is frequently stiff and fibrotic, impeding the diffusion of PS molecules and oxygen, while impaired perfusion further limits nutrient and oxygen delivery. Light scattering and absorption in biological tissues restrict penetration depth, confining PDT efficacy to superficial lesions. Finally, variability in PS uptake across tumour regions leads to uneven ROS generation, leaving residual disease that can drive relapse [37,40].

#### 2.3.1. Tumour Hypoxia and Oxygen Deprivation

Hypoxia, defined as a local reduction in oxygen availability, is a hallmark of solid tumours and a major barrier to the effectiveness of PDT [41]. The oxygen dependence of conventional PDT, particularly Type II pathways that generate singlet oxygen, makes it highly sensitive to the oxygenation status of the tumour. In hypoxic regions, the limited availability of molecular oxygen directly reduces ROS formation, leading to incomplete tumour cell killing and potential treatment resistance. Tumour hypoxia arises from several structural and functional abnormalities within the TME. Tumour blood vessels are often disorganised, tortuous, and leaky, resulting in uneven perfusion and insufficient oxygen delivery. Rapid tumour growth further increases oxygen demand, as proliferating cells outpace the oxygen supply, particularly in the tumour core. Elevated metabolic activity further depletes oxygen, as tumour cells consume oxygen at rates faster than diffusion can replenish. In addition, high interstitial fluid pressure impairs blood flow and oxygen diffusion, creating pockets of severe oxygen limitation [42,43,44].

In Type II PDT, singlet oxygen is produced only in the presence of molecular oxygen, so hypoxic regions experience drastically reduced cytotoxicity [8,45]. Although Type I PDT is less oxygen-dependent, its efficiency also declines under hypoxic conditions as reduced ROS generation limits the overall oxidative stress required for effective tumour destruction. Hypoxia also triggers adaptive responses within tumour cells, including the stabilization of hypoxia-inducible factors (HIFs) [30,45,46,47]. These transcription factors promote angiogenesis, metabolic reprogramming, and resistance to apoptosis, further protecting tumour cells from PDT-induced damage and contributing to aggressive growth and metastatic potential [47,48].

#### 2.3.2. Antioxidant Defences and Redox Buffering

Tumours possess a highly developed antioxidant defence system that counteracts the cytotoxic effects of PDT-generated ROS. Cancer cells typically exhibit elevated levels of intracellular antioxidants such as glutathione, thioredoxin, and superoxide dismutase, which collectively neutralise singlet oxygen and free radicals before they can induce irreversible oxidative damage. This heightened redox buffering capacity is driven by chronic metabolic stress within tumours and is further supported by hypoxia-inducible signalling, which upregulates antioxidant enzymes through pathways such as Nrf2 [49,50]. As a result, even when PDT successfully generates ROS, a significant portion are rapidly quenched, thereby reducing treatment efficiency. The presence of high GSH concentrations within the TME also inactivates many PS molecules and oxygen-generating nanomaterials through unwanted redox reactions. Consequently, the antioxidant landscape of tumours creates a major barrier that limits ROS accumulation and weakens downstream apoptotic and necrotic pathways initiated during PDT [49,50,51].

#### 2.3.3. Structural and Transport Barriers Within the Tumour Microenvironment

The physical architecture of solid tumours imposes significant constraints on effective PDT. Tumours develop a dense and disorganised ECM composed of collagen, fibronectin, and hyaluronic acids, which restricts the penetration and uniform distribution of PS molecules. Abnormal vasculature further contributes to uneven delivery by creating regions of poor perfusion, where therapeutic agents accumulate insufficiently [52,53]. High interstitial fluid pressure (IFP), a hallmark of many aggressive tumours, opposes convective transport and hinders the movement of both nanomaterials and molecular PSs from blood vessels into deeper tumour zones [54,55]. The structural barriers create spatial variability in PS concentration, producing under-treated regions that survive PDT and may contribute to recurrence. Restricted oxygen diffusion within this microenvironment compounds the challenge by slowing replenishment of oxygen required for sustained ROS generation. Together, these architectural and transport limitations reduce PDT uniformity and overall therapeutic success [52,56].

#### 2.3.4. Light Attenuation and Heterogeneous Photosensitizer Distribution

Effective PDT requires uniform light delivery throughout the tumour, yet light penetration in biological tissues is inherently limited. Optical scattering and absorption by haemoglobin, water, and cellular components cause progressive attenuation of light intensity as it travels through tissue, resulting in steep depth-dependent reductions in photon availability [8,25]. While NIR wavelengths improve penetration, most solid tumours still receive limited light irradiation, with deeper regions exposed to sub-therapeutic light doses [57]. This limitation becomes more pronounced when the PS distribution is also heterogeneous, as is typical in tumours with poor vascularization and high IFP. Consequently, some tumour areas receive both insufficient light and inadequate PS concentrations, preventing efficient ROS generation. These untreated regions allow tumour cells to survive and contribute to incomplete tumour eradication. Light attenuation, therefore, represents a critical physical constraint that compounds the biochemical and structural barriers already present in the TME [8,25,58].

## 3. Nanoenzyme-Enhanced Catalytic Photodynamic Therapy (CAT-PDT)

While PDT is recognised for selective cytotoxicity against cancer cells, its therapeutic success is limited by intrinsic TME barriers. Factors such as hypoxia, elevated antioxidant capacity, poor PS penetration, and limited tissue light penetration collectively reduce ROS formation during PDT. These limitations are intrinsic to solid tumours and cannot be fully corrected by adjusting conventional PDT parameters such as increasing light penetration, increasing PS concentration, or altering incubation times. As a result, PDT often produces uneven treatment responses, with superficial or well-oxygenated regions responding more effectively than deeper, hypoxic, or antioxidant-rich regions [56,59].

Nanoenzyme-enhanced catalytic photodynamic therapy (CAT-PDT) was therefore developed to directly address these TME-mediated limitations. In contrast to conventional PDT, which relies on externally delivered oxygen, light, and PS molecules, CAT-PDT introduces catalytic components that modulate biochemical conditions within the tumour. These systems can locally generate oxygen, disrupt redox buffering, amplify ROS production through alternative pathways, or improve the transport and distribution of therapeutic agents within the tumour. The result is a form of PDT that is less dependent on uniform oxygenation, less vulnerable to antioxidant upregulation, and more capable of operating effectively across the heterogeneous and complex architecture of solid tumours [12,60,61,62]. 

### 3.1. Historical Development of Catalytic Photodynamic Therapy

The foundation of CAT-PDT lies in the field of nanoenzyme research, which began with the introduction of the term “nanoenzyme” in 2004 to describe nanomaterials with intrinsic ribonuclease-like properties (Table 2) [63,64]. A pivotal moment occurred in 2007 when ferromagnetic nanoparticles were discovered to possess peroxidase-like activity, enabling them to catalyse the production of ROS from H_2_O_2_ [65]. This discovery opened the door to using nanoenzymes as catalytic agents in cancer therapy. A critical milestone for this review was the subsequent integration of these nanoenzymes with PDT around 2018, when researchers reported Pt nanoenzymes decorated on PS-MOFs for enhanced PDT [66]. Early CAT-PDT systems focused on catalase-like nanoenzymes to generate O_2_ from endogenous H_2_O_2_, directly addressing the problem of tumour hypoxia that limits conventional PDT. This evolved into the use of peroxidase/Fenton-like nanoenzymes to produce •OH independently of oxygen. By the late 2010s, the field advanced to more sophisticated cascade systems that combine multiple catalytic activities to sustain ROS generation across the heterogenous TME [67,68].

**Table 2:** Historical development of CAT-PDT.

Period	Key Development	Scientific Focus	Emergence in Cancer Therapy	Limitations Addressed	Key References
2004	Term “nanoenzyme” coined	AuNPs with ribonuclease-like activity	Not yet applied to cancer therapy	N/A; foundational discovery	[64]
2007	Discovery of peroxidase-like activity in Fe_3_O_4_ nanoparticle_s_	POD and OXD mimics	Early exploration of pro-oxidative nanoenzymes for therapy	Need stable, cost-effective enzyme substitutes	[65]
~2018	Integration of nanoenzymes with photosensitisers; CAT-PDT concept established	Catalase-like O_2_ generation; peroxidase-like •OH generation	First demonstrations of enhanced PDT efficacy in vitro and in vivo	Hypoxia; oxygen-dependent Type II PDT; limited ROS	[69]
2020-present	Development of cascade catalytic systems and multifunctional nanoenzymes	Sequential catalysis; tumour responsive systems	Synergistic therapies; overcoming complex TME	Substrate heterogeneity; spatial ROS variability; poor PS delivery	[67]

### 3.2. Mechanisms of CAT-PDT

**Figure 3:**
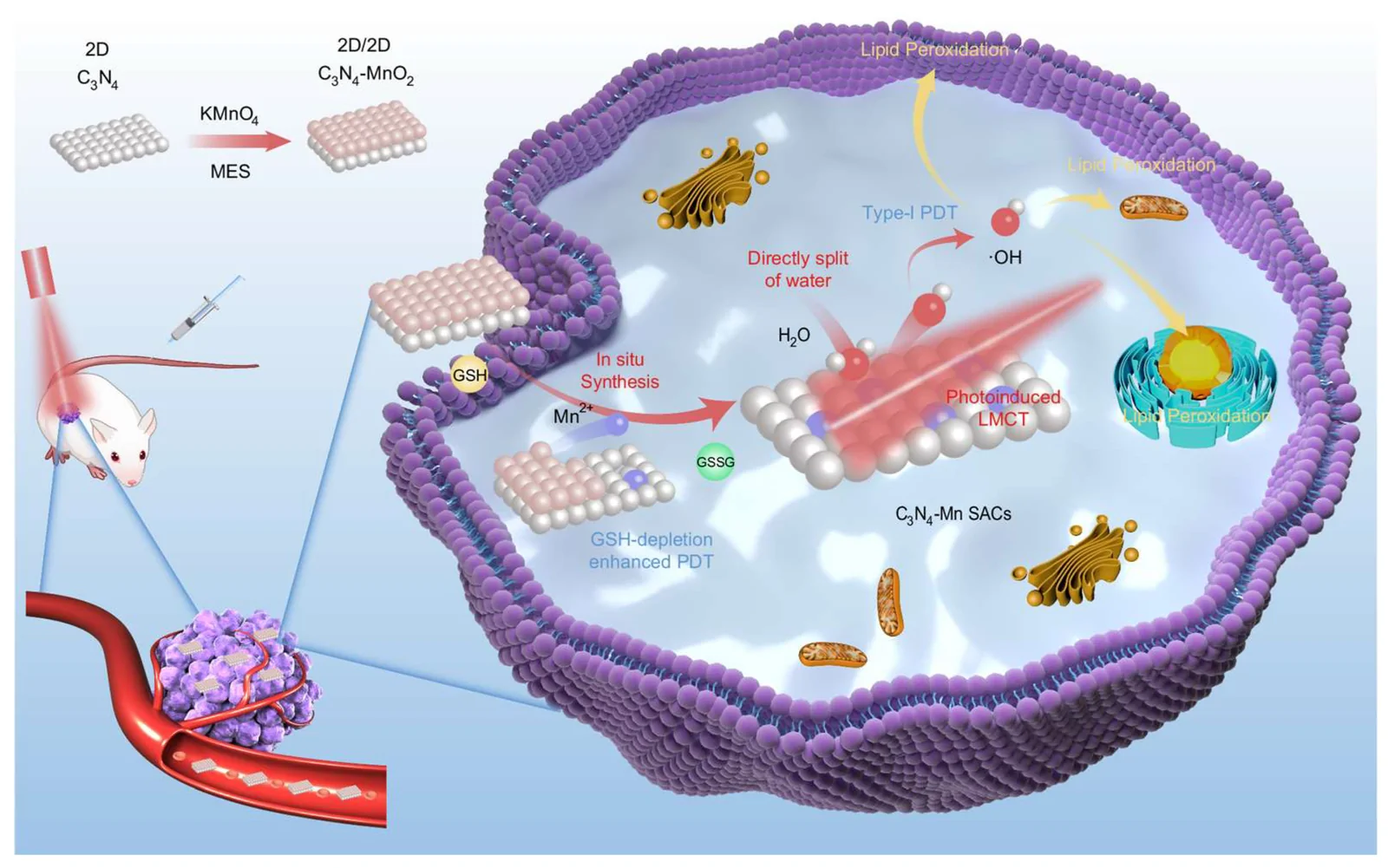
Representative example of oxygen-independent CAT-PDT. GSH in the tumour microenvironment reduces C_3_N_4_-MnO_2_, releasing Mn^2+^ that coordinates with C_3_N_4_ to form C_3_N_4_-Mn single-atom catalysts. Under 660 nm light, these single-atom catalysts generate •OH through water-splitting without requiring O_2_ or H_2_O_2_ inducing lipid peroxidation and cell death. Adapted from [81] under CC BY 4.0 (https://creativecommons.org/licenses/by/4.0/). https://www.nature.com/articles/s41467-024-46987-1 (accessed 2 October 2026).

CAT-PDT integrates classical PDT reactions with catalytic processes that enhance intracellular ROS generation [70,71]. This mechanism initiates when the PS absorbs photons and transitions from the ground state to an excited singlet state, followed by intersystem crossing to a long-lived triplet state. In conventional PDT, this triplet state transfers energy to molecular oxygen to generate singlet oxygen or undergoes electron transfer to form superoxide and downstream ROS (Figure 3) [31]. CAT-PDT extends these pathways by incorporating catalytic nanoparticles or enzyme-mimetic systems capable of continuously converting endogenous substrates into ROS, thereby sustaining cytotoxic oxidative pressure even in hypoxic regions [12,71].

A central mechanism involves Fenton or Fenton-like catalysis, in which metal-based nanomaterials such as Fe, Cu, or Mn species convert intracellular H_2_O_2_ into hydroxyl radicals [70,72,73]. This process operates independently of external oxygen delivery and is therefore effective in the hypoxic microenvironment typical of solid tumours. This catalytic activity is often enhanced by the elevated H_2_O_2_ concentrations and acidic pH of tumour tissues, which increase metal ion redox cycling and accelerate ROS generation. The photogenerated electrons from the excited PS can additionally drive metal-ion reduction, maintaining the catalytic cycle and amplifying ROS production under light activation [73,74,75].

It is important to distinguish between two mechanistically distinct modes of CAT-PD. In oxygen-generating CAT-PDT, catalase-like nanoenzymes disproportionate H_2_O_2_ into O_2_ and H_2_O_2,_ relieving hypoxia and sustaining Type II photochemistry [76]. In oxygen-independent CAT-PDT, peroxidase/Fenton-like nanoenzymes convert H_2_O_2_ into •OH through redox cycling. This reaction is not inherently light-dependent as •OH is generated directly from H_2_O_2_ and does not require photochemical activation. In CAT-PDT systems, however, photogenerated electrons can reduce metal ions, accelerating the Fenton cycle and creating a synergistic, light-enhanced catalytic effect [73,74,75].

The mechanism of CAT-PDT relies on oxygen microenvironment modulation, where catalytic nanomaterials disproportionate endogenous H_2_O_2_ into molecular oxygen, increasing the local oxygen concentration available for Type II photochemical reactions [76]. This process modulates PDT efficiency in hypoxic tumours and promotes simultaneous Type I and Type II pathways. In addition, certain catalytic platforms can exhibit oxidase-like or peroxidase-like activity, promoting ROS production in the absence of light irradiation. However, in the presence of light, these catalytic activities improve electron transfer [77,78]. This dual-mode activity produces a continuous and localised ROS supply that exceeds the transient levels observed in conventional PDT. Overall, CAT-PDT utilises synergistic interactions between photochemical excitation and catalytic redox systems to overcome hypoxia, increase ROS yield, and improve therapeutic efficacy. The integration of catalytic cycles ensures sustained ROS accumulation, amplification of oxidative injury, and enhanced induction of apoptosis and necrosis in tumour cells [79,80].

### 3.3. Nanoenzymes as Catalytic Drivers of CAT-PDT

**Figure 4:**
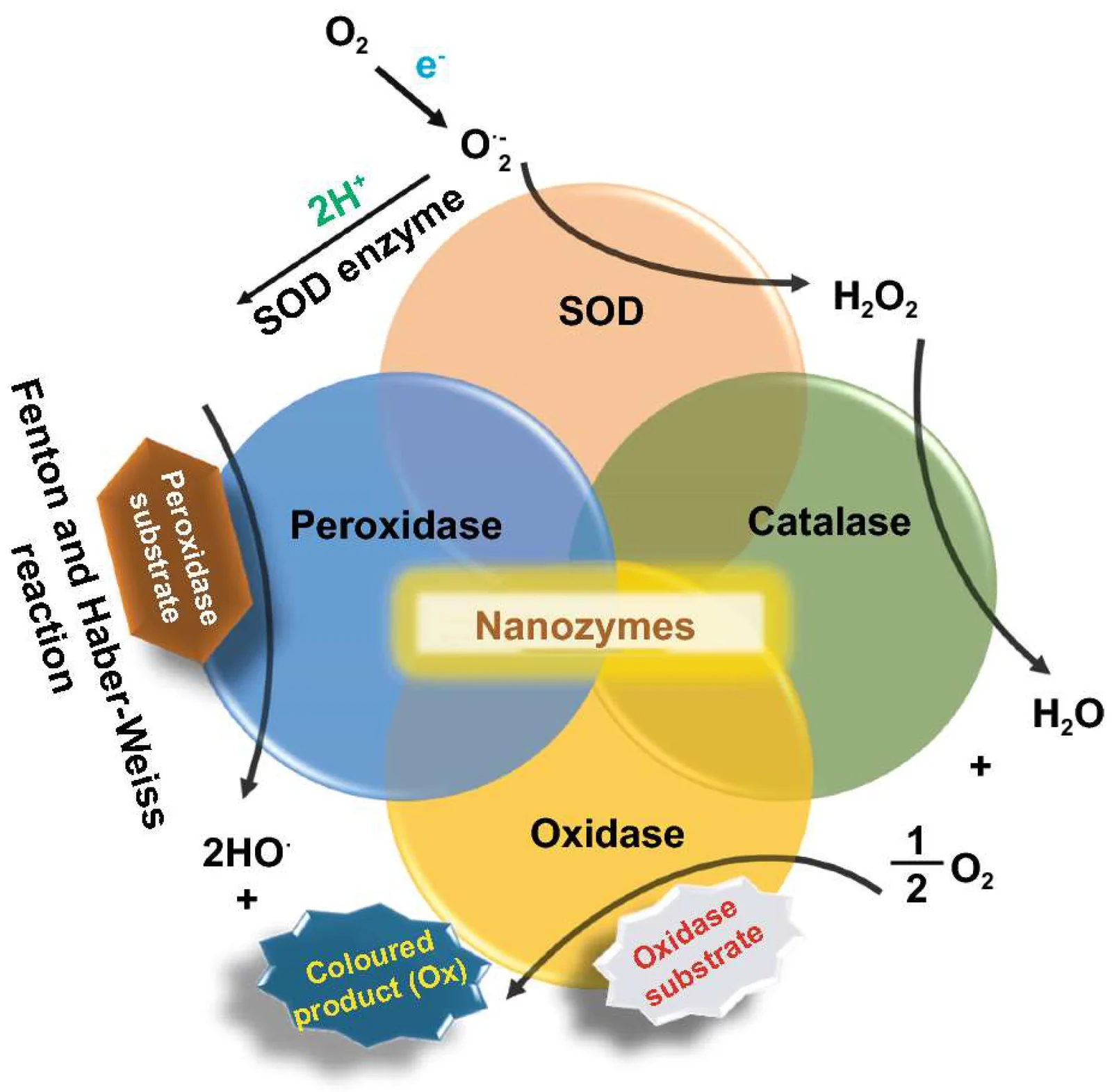
Overview of nanoenzyme catalytic activities relevant to catalytic photodynamic activity. Nanoenzymes can exhibit multiple enzyme-like activities, including catalase-, peroxidase-, oxidase-, and SOD-like activities. SOD-like catalyses the dismutation of SOD radicals (O_2_•^−^) into H_2_O_2_ and O_2 (_2 O_2_•^−^ + 2H^+^ → H_2_O_2_ + O_2_) catalase-like activity disproportionates H_2_O_2_ into H_2_O and O_2_ (2H_2_O_2_ → 2H_2_O + O_2_) peroxidase-like activity converts H_2_O_2_ into highly cytotoxic hydroxyl radicals (•OH) through Fenton and Harber–Weiss chemistry (H_2_O_2_ + substrate → •OH or oxidised products). Oxidase-like activity reduces molecular O_2_ into H_2_O_2_ using various substrates (Substrate + O_2_ → Product + H_2_O_2_). Adapted from [84] under CC BY 4.0 (https://creativecommons.org/licenses/by/4.0/, accessed on 20 August 2026).

Nanoenzymes are central to CAT-PDT due to their ability to introduce enzyme-mimetic activities that extend the oxidative capacity of conventional PDT. In contrast to natural enzymes, nanoenzymes are stable under tumour-associated conditions, such as low pH and elevated H_2_O_2_, and can be engineered for controlled catalytic behaviour. Their functions include oxygen regeneration, ROS amplification, and modulation of antioxidant defences, all of which address the biochemical limitations of the TME. In CAT-PDT, nanoenzymes act as catalytic drivers that sustain ROS generation even in hypoxic or antioxidant-rich regions. By disproportionating H_2_O_2_, generating hydroxyl radicals, or converting superoxide into secondary ROS, they maintain oxidative stress across heterogeneous tumour sites. This catalytic reinforcement transforms PDT from a strictly oxygen-dependent therapy into a dual-modality system where photochemical and catalytic processes operate in synergy. Nanoenzymes can exhibit multiple enzyme-like activities simultaneously, including SOD-like, catalase-like, peroxidase-like, and oxidase-like activities. These activities are mechanistically interconnected, with H_2_O_2_ serving as a central intermediate that links the catalytic pathways. The balance between these activities determines the overall ROS profile and therapeutic efficacy of CAT-PD. Figure 4 provides an overview of these interconnected catalytic cycles and their contributions to ROS generation [22,50,81,82,83,84].

The following table (Table 3) and subsections outline the major classes of nanoenzymes, catalase-like, peroxidase-like, oxidase-like, superoxide dismutase-like, multifunctional cascade systems, and metal–organic or hybrid biomimetic systems, highlighting their specific catalytic roles and contributes the efficacy of CAT-PDT [22,50,82,83,84].

#### 3.3.1. Catalase-like Nanoenzymes

Catalase-like nanoenzymes are the most widely applied systems in CAT-PDT due to their ability to disproportionate endogenous H_2_O_2_ into molecular oxygen (2H_2_O_2_ → 2H_2_O + O_2_) [85,86], thereby replenishing the intratumoral oxygen pool required for PS activation [87]. However, the effectiveness of catalase-like nanoenzymes in vivo depends on the local availability of H_2_O_2_, which is heterogenous across tumours and may differ substantially between experimental models and individual tumour regions [88]. Consequently, high catalase-like activity measured using exogenous or relatively high concentrations of H_2_O_2_ in vitro may not necessarily correspond to equivalent oxygen generation in the TME. By generating oxygen from H_2_O_2_, catalase-like nanoenzymes can increase local oxygen availability and potentially support oxygen-dependent photochemical reactions during irradiation; however, the extent of this effect depends on substrate availability, catalytic efficiency, and nanoenzyme localisation within the tumour [87,88].

The activity of catalase-like nanoenzymes is enhanced under the mildly acidic pH of tumour tissues, which favours catalytic turnover compared to physiological conditions. By supplying a transient, substrate-limited flux of oxygen, catalase-like nanoenzymes sustain singlet-oxygen production during irradiation, provided sufficient H_2_O_2_ is available. Endogenous H_2_O_2_ concentrations in tumour cells are typically in the low molecular range, and once H_2_O_2_ is depleted, O_2_ generation ceases unless H_2_O_2_ is replenished by cellular metabolism or external sources. This catalytic oxygen regeneration reduces dependence on vascular oxygen delivery, stabilises redox balance, and provides the biochemical foundation for other catalytic pathways. Materials such as MnO_2_ nanoparticles, cerium oxide, and certain MOFs have demonstrated strong catalase-mimetic behaviour and have been validated as effective PDT amplifiers [86,89,90].

#### 3.3.2. Peroxidase-like Nanoenzymes

Peroxidase-like nanoenzymes contribute to CAT-PDT by converting H_2_O_2_ into hydroxyl radicals through redox cycling reactions. This activity proceeds independently of light, enabling continuous ROS generation even in the absence of irradiation. When co-localised with PS molecules, peroxidase-like nanoenzymes create a dual-ROS environment in which hydroxyl radicals complement photodynamically generated singlet oxygen. Hydroxyl radicals are highly reactive and short-lived, allowing rapid damage to biomolecules without reliance on oxygen diffusion or PS distribution. This catalytic behaviour is particularly advantageous in tumour regions with shallow light penetration or heterogeneous PS accumulation [90,91,92].

However, peroxidase-like activity remains dependent on the availability of H_2_O_2_ and suitable reaction conditions within the TME. Consequently, catalytic activity measured under optimised in vitro conditions may not directly predict ROS generation in vivo, where substrate concentrations, pH, intracellular localisation, and nanoparticle distribution are heterogenous [12,93]. In addition, as hydroxyl radicals are highly reactive and short-lived, their therapeutic effect is likely to depend strongly on the spatial proximity of the nanoenzyme to that target site [12,87,93].

#### 3.3.3. Oxidase-like Nanoenzymes

Oxidase-like nanoenzymes generate superoxide species by catalysing the reduction of oxygen using tumour-associated metabolites as electron donors. In contrast to peroxide-like systems, they do not require H_2_O_2_ as a substrate, which allows them to function in tumour regions where peroxide levels fluctuate. The superoxide produced contributes directly to oxidative stress and undergoes dismutation to yield H_2_O_2_, which can then be exploited by catalase- or peroxidase-like nanoenzymes. This catalytic network enhances ROS diversity and abundance, creating a more oxidative environment that sensitizes tumour tissues to photodynamic damage [90,91,94].

However, oxidase-like activity is dependent on both molecular oxygen and the availability of suitable electron-donor substrates, which may limit its contribution in severely hypoxic or metabolically heterogenous tumour regions. The extent of ROS generation observed in vitro may therefore not reflect activity in vivo, where oxygen availability, substrate concentrations, and nanoenzyme localisation can vary substantially [95,95].

#### 3.3.4. Superoxide Dismutase (SOD)-like Nanoenzymes

Superoxide dismutase (SOD)-like nanoenzymes catalyse the conversion of superoxide radicals into H_2_O_2_ and oxygen. While this function is protective in normal physiology, in CAT-PDT, it increases the availability of H_2_O_2_ as a substrate for other catalytic pathways. SOD-like activity therefore acts as an upstream modulator, routing superoxide into ROS forms that are more effective for tumour ablations. When integrated into CAT-PDT systems, these nanoenzymes stabilise ROS flux and maintain catalytic turnover, particularly when combined with catalase- or peroxidase-like systems [90,91,94].

However, the contribution of SOD-like activity to CAT-PDT is context-dependent. Although conversion of superoxide to H_2_O_2_ may provide a substrate for downstream catalytic reactions, increased H_2_O_2_ availability does not necessarily translate into greater PDT efficacy and may instead contribute to oxidative stress or cellular antioxidant responses [88,96]. The therapeutic outcome therefore depends on the balance between superoxide conversion, downstream H_2_O_2_ utilization, and the capacity of the TME to buffer the resulting ROS [88,93].

#### 3.3.5. Multi-Enzyme and Cascade-Catalytic Nanoenzymes

Multifunctional nanoenzymes combine two or more catalytic behaviours within a single nanoplatform enabling sequential or parallel reactions. For example, catalase-peroxidase hybrids simultaneously regenerate oxygen and produce hydroxyl radicals, ensuring that oxygen supply and ROS generations occur in tandem. Cascade-catalytic architectures improve efficiency by positioning sequential reactions in proximity, reducing diffusion distances for substrates and intermediates. This integration makes multi-enzyme nanoenzymes among the most efficient catalysts in CAT-PDT, capable of sustaining oxidative stress in heterogeneous tumour regions and under fluctuating substrate concentrations [82,90].

Despite these advantages, cascade systems also introduce additional variables that can affect therapeutic performance. Each catalytic step requires appropriate substrate availability and reaction conditions; thus, efficient activity of one enzyme-like component does not necessarily ensure efficient operation of the complete cascade in vivo. Differences in nanoenzyme distribution, substrate concentration, reaction kinetics, and tumour heterogenicity may therefore reduce cascade efficiency compared with results obtained under controlled in vitro conditions [97,98,99].

#### 3.3.6. Metal–Organic and Hybrid Biomimetic Nanoenzymes

Metal–organic nanoenzymes, including frameworks and complex nanomaterials, provide tuneable catalytic systems with high surface areas and the ability to incorporate PS molecules directly into their structures. Their activity can be tailored through the choice of metal, they design of the ligand, pore architecture, and surface functionalization, enabling precise control over oxygen generation, ROS production, and redox modulation. Organic–inorganic hybrid nanoenzymes extend this versatility by combining catalytic metals with biocompatible organic components, creating defined catalytic microenvironments that facilitate substrate access and controlled ROS generation. Their modulatory and programmability make them highly adaptable to CAT-PDT systems requiring simultaneous control of catalytic, photochemical, and biological processes [66,90,100].

However, the structural versatility of these systems also introduces considerable variability in catalytic behaviour. Changes in metal centres, ligand environment, functionalization, pore structure, and PS loading can alter substrate accessibility and catalytic activity, making direct comparison between different MOF-based and hybrid systems challenging. Moreover, the enhanced catalytic performance observed in engineered nanoplatforms may not translate proportionally to therapeutic efficacy in vivo because tumour accumulation, protein interactions, biodegradation, and heterogenous substrate availability may influence their behaviour [88,93].

## 4. Applications of Nanoenzyme-Based CAT-PDT

The advancement of CAT-PDT depends on whether nanoenzymes can overcome the biochemical limitations of conventional PDT in real time, particularly hypoxia, poor ROS generation, and redox saturation. Published studies increasingly demonstrate that nanoenzyme systems can dynamically modulate the tumour microenvironment during irradiation, sustain oxygenation, amplifying verified real-time outcomes, providing direct evidence that catalytic modulation translates into therapeutic improvement [100,101].

### 4.1. Catalytic Oxygen Generation During CAT-PDT

Recent studies show that nanoenzymes rapidly disproportionate endogenous H_2_O_2_ into O_2_ during irradiation, directly supporting PS excitation. Systems based on MnO_2_, Au, Pt-alloys, and multi-enzyme MOFs have reported transient, substrate-limited oxygen evolution visualised using dissolved oxygen probes, hypoxia-sensitive dyes, and oxygen-mapping imaging [71,83]. In one study, researchers developing an Au@ZIF-8 nanoplatform demonstrated that AuNPs incorporated into ZIF-8 exhibited catalase-like activity and promoted H_2_O_2-_dependent O_2_ generation, resulting in enhanced singlet oxygen production and PDT efficacy [102]. Similarly, Lin and colleagues [103] developed an MnO_2_-based oxygen-generating system, HSA-MnO_2_-Ce6, to alleviate tumour hypoxia and restore oxygen availability for PDT. The system generated O_2_ in response to endogenous H_2_O_2_ and increased singlet oxygen generation under irradiation. In an orthotopic bladder cancer model, administration of HSA-MnO_2_-Ce6 increased tumour O_2_ content and resulted in improved PDT efficacy and prolonged survival, providing in vivo evidence that catalytic oxygen generation can translate into therapeutic benefit [103].

Pt-based nanoenzymes have similarly been investigated for overcoming oxygen limitation. In one study, researchers loaded a PS molecule with Pt nanoparticles exhibiting catalase-like activity. The Pt nanoenzyme catalysed H_2_O_2_-dependent O_2_ generation, facilitating singlet oxygen formation under hypoxic conditions and enhancing the photodynamic effect of the MOF nanoplatform [69]. Cai and colleagues [104] subsequently incorporated Pt nanoparticles into three-dimensional dendritic mesoporous silica nanoparticles loaded with Ce6. The Pt nanoparticles catalysed intracellular H_2_O_2_ to generate O_2_, while mitochondrial targeting with triphenylphosphine promoted localisation of the system. The resulting platform enhanced intracellular ROS generation and increased apoptosis in A549 lung cancer cells, demonstrating the potential of combining catalytic oxygen generation with PS delivery and subcellular targeting [104].

The oxygen-supplying strategy has also been extended to multifunctional nanoenzyme systems. In a recent study, researchers engineered an oxygen-supplying haemoglobin-integrated nanoenzyme encapsulating curcumin. The system alleviated tumour hypoxia while sustaining ROS generation, resulting in synergistic catalytic and photodynamic efficacy in breast cancer models. The study demonstrated tumour regression and the potential of multifunctional nanoenzymes for overcoming hypoxia and enhancing PDT in solid tumours [99].

These studies demonstrate that catalytic oxygen generation can enhance PDT across various nanoenzyme-based platforms. This evidence extends beyond measurements of oxygen generation to increased singlet oxygen production, enhanced tumour destruction, tumour suppression, and improved survival in animal models. Despite this, the magnitude of the therapeutic efficacy remains dependent on factors including endogenous H_2_O_2_ availability, nanoenzyme distribution, catalytic efficiency, and oxygen consumption during light irradiation [69,102,104,105,106].

### 4.2. ROS Amplification Under Catalytic Conditions

CAT-PDT requires both an oxygen supply and ROS turnover in catalase-like systems, whereas peroxidase-like systems can generate ROS independently of O_2_. Real-time ROS tracking using DCFH-DA, SOSG, or ESR quantification consistently shows that nanoenzymes with POD-like activity produce significantly increased ROS flux compared to conventional PDT [107,108]. Studies involving Fe_3_O_4_, Cu-based nanoenzymes, CeO_2_ redox-cycling systems, and peroxidase-active MOFs demonstrate that catalytic pathways sustain oxygen and oxidised byproduct generation during light irradiation, confirming that ROS amplification occurs during the treatment window itself [109,110].

In another study, researchers developed Fe_3_O_4_-Hb@cur magnetic bead nanoenzymes capable of exemplary ROS amplification by combining Fenton reaction-mediated generation of hydroxyl radicals with haemoglobin-supplied oxygen under hypoxic conditions. Following NIR irradiation, the available oxygen supported singlet oxygen generation by curcumin, while the catalytic activity of Fe_3_O_4_ contributed additional ROS. The researchers reported increased intracellular ROS levels and demonstrated that this combined oxidative stress produced substantial cytotoxicity in breast cancer cells and inhibited tumour growth in vivo [106].

Similar catalytic amplification has been achieved using multifunctional nanoenzymes capable of multiple enzyme reactions. In a recent study, Fe_3_O_4_/Ag/B_i2_MoO_6_ nanoparticles were designed with peroxidase-, catalase-, superoxide-dismutase, and oxidase-like activities in combination with photodynamic activity. The coordinated catalytic reactions were designed to maintain ROS production within the TME while simultaneously regulating oxygen and antioxidant levels, resulting in synergistic chemodynamic, photodynamic, and photothermal therapy [111].

Collectively, these studies indicate that CAT-PDT can increase oxidative stress through complementary catalytic and photodynamic pathways instead of relying solely on singlet oxygen generation by PS molecules. This provides a mechanistic basis for the enhanced cytotoxicity observed with CAT-PDT systems. However, increased ROS generation does not necessarily translate into therapeutic efficacy, and the balance between ROS generation and scavenging within the TME can influence the final response [106].

### 4.3. Monitoring of Nanoenzyme-PS Co-Localisation

Imaging techniques, including fluorescence microscopy, photoacoustic imaging, and MRI, can be used to investigate the distribution and co-localisation of nanoenzymes and PS molecules within tumour tissue [112]. Demonstrating that both therapeutic components accumulate within the same tumour regions provides spatial evidence that catalytic and photodynamic functions can occur within a shared spatial microenvironment. In a recent study, a self-assembled single-atom ruthenium nanoenzyme conjugated onto a Mn_3_(Co(CN)_6_)_2_ metal–organic framework was co-loaded with Ce6. The integrated platform enabled catalase-like O_2_ generation through the reaction of endogenous H_2_O_2_ with the single atom Ru sites, while MRI was used to track the accumulation of the nanoplatform within tumour tissue. This integrated design relieved tumour hypoxia and enhanced ROS generation, improving PDT efficacy in vivo [101].

Similarly, a Pt-Ce6 nanoplatform consisting of porous Pt nanoparticles functionalised as catalase-like nanoenzymes and Ce6 was developed. The Pt-mediated catalytic conversion of endogenous H_2_O_2_ generates oxygen, modulating hypoxia, while Ce6 provides the PS molecule for the PDT component and NIR-II photothermal imaging can be used to monitor the efficacy, distribution, and accumulation of the nanoplatform within tumour tissue, supporting localised delivery of the catalytic and photodynamic components [113].

### 4.4. Catalytic Responses in the TME

CAT-PDT can mediate the TME by simultaneously modulating tumour hypoxia, redox balance, ROS availability. Several studies have demonstrated how catalytic nanoenzymes simultaneously relive hypoxia, disrupt antioxidant buffering, and sustain ROS flux across heterogeneous tumour regions [97]. In one study, MnO_2_ nanosheets were integrated into a biomimetic nanoplatform and shown to disproportionate endogenous H_2_O_2_ into oxygen, thereby restoring singlet oxygen generation in hypoxic tumour cores. In vivo imaging demonstrated improved therapeutic efficacy, with greater uniformity and significantly enhanced tumour regression compared with conventional PDT controls [114].

Another study employed cerium oxide nanoparticles with SOD- and catalase-like activities to regulate H_2_O_2_ metabolism within the TME. The SOD-like activity converted superoxide radicals into H_2_O_2_, while the catalase-like activity subsequently converted H_2_O_2_ into oxygen, increasing local oxygen availability and mediating tumour hypoxia. When combined with Ce^4+^and glucose oxidase, this dual regulation of H_2_O_2_ demonstrated enhanced oxygen and improved the efficacy of PDT, demonstrating how catalytic manipulation of endogenous substrates can transform the TME to support PDT [115].

Together, these findings highlight that CAT-PDT is not a transient intervention by a dynamic process that continuously remodels the TME. By sustaining oxygen generation, amplifying ROS, and depleting buffers, catalytic nanoenzymes transform the tumour niche from a protective environment into one highly vulnerable to oxidative destruction. The persistent catalytic activity ensures deeper penetration, more uniform therapeutic distribution, and ultimately, more durable treatment [16].

## 5. Limitations, Pharmaceutical Development, and Clinical Translation

Despite its promise, CAT-PDT faces several challenges and limitations. These limitations do not undermine the therapeutic potential of CAT-PDT but highlight critical areas requiring refinement before clinical application can be achieved. This section outlines the major barriers affecting nanoenzyme performance, stability, selectivity, and real-world feasibility [116,117].

### 5.1. TME Heterogeneity and Inconsistent Catalytic Performance

Nanoenzyme catalysis depends on environmental factors such as pH, H_2_O_2_ concentration, oxygen availability, and redox balance. However, these parameters vary substantially across tumour types and even within different regions of the same tumour. As a result, nanoenzymes may perform efficiently in one TME but sub-optimally in another. Catalase-like activity requires sufficient endogenous H_2_O_2_, whereas peroxidase-like activity accelerates under acidic conditions [118]. Thus, tumours that do not meet these biochemical thresholds show weaker catalytic responses. This heterogeneity may contribute to spatially variable ROS generation and PDT efficacy, particularly in poorly perfused or metabolically distinct tumour regions. In addition, heterogeneity creates inconsistency in therapeutic outcomes and complicates the prediction of catalytic efficiency across patient populations [97,119].

Catalytic performance measured under controlled in vitro conditions may not accurately predict activity in vivo, where pH, substrate concentrations, oxygen availability, and redox status vary spatially within the tumour. This highlights the need for physiologically relevant and standardised approaches for evaluating nanoenzyme activity under conditions that reflect the heterogenous TME [118].

### 5.2. Physiological Properties, Colloidal Stability, and Protein Corona Formation

Physiological properties such as particle size distribution, surface charge, morphology, and surface functionalisation can influence the colloidal stability, biodistribution, cellular uptake, and clearance or nanoenzyme-based CAT-PDT systems [120]. Aggregation of particles may further alter hydrodynamic size, catalytic-site accessibility, and biological distribution, making colloidal stability an important quality attribute for clinical development [121]. Following administration, adsorption of plasma proteins can form a dynamic protein corona that changes nanoenzyme surface properties, cellular interactions, pharmacokinetics and catalytic behaviour [121].

Most CAT-PDT systems depend on endogenous H_2_O_2_ as the catalytic substrate driving oxygen production or ROS generation. However, H_2_O_2_ levels vary widely across tumours, and in many cases, they may be insufficient to sustain continuous catalysis. This reliance creates a bottleneck that restricts nanoenzyme activity and may lead to incomplete therapeutic effects, especially in deeper tumour regions where metabolic gradients limit H_2_O_2_ production [118,122].

### 5.3. Potential for Excessive or Uncontrolled ROS Generation

While ROS amplification is the central objective of CAT-PDT, excessive or uncontrolled ROS generation raises concerns regarding off-target oxidative stress, collateral damage to surrounding tissues, inflammatory activation, and potential long-term toxicity. In complex biological systems, ROS diffusion and catalytic propagation are difficult to strictly localize. Although most studies report favourable safety profiles, the possibility of uncontrolled catalytic cascades remains a key translational barrier. More advanced regulatory controls and self-limiting catalytic designs are needed [56,123].

### 5.4. Manufacturing Complexity and Scalability Challenges

The translation from CAT-PDT is hindered by the manufacturing complexity of the nanoenzyme-based systems. Many reported systems rely on multi-component architectures, defect engineering, or biomimetic surface modifications, which complicate large-scale synthesis and reproducibility. Small variations in fabrication parameters can significantly alter catalytic activity, PS loading, and biological behaviour, leading to batch-to-batch inconsistency. For clinical translation, scalable, reproducible, and simplified nanoenzyme designs that maintain catalytic performance will be essential to meet manufacturing and quality-control requirements [124,125].

Critical quality attributes should include particle-size distribution, surface characteristics, PS loading and release, catalytic activity, purity, colloidal stability, storage stability, and batch-to-batch reproducibility. Manufacturing processes must also demonstrate compatibility with sterilization and endotoxin control requirements to ensure consistent clinical-grade production [126,127].

### 5.5. Regulatory Considerations and Clinical Translation

Regulatory approval represents a major challenge for CAT-PDT due to its hybrid nature, combining PS molecules, catalytically active nanomaterials, and light-delivery devices. The presence of catalytically active nanoenzymes raises concerns regarding long-term safety, biodistribution, and potential off-target ROS generation. In addition, the lack of standardised regulatory frameworks for catalytic nanotherapies complicates clinical evaluation and approval pathways. Successful translation will require controllable catalytic behaviour, comprehensive safety assessments, and alignment with existing clinical PDT protocols to facilitate regulatory acceptance [125,128].

A primary concern for clinical translation is the long-term biosafety of nanoenzymes. Unlike traditional PS molecules, nanoenzymes often contain redox-active metals or complex hybrid architectures that may persist in vivo. Prolonged retention of catalytic nanomaterials within organs such as the liver, spleen, and kidneys raises concerns regarding chronic oxidative stress, inflammation, and potential organ dysfunction. Additionally, the intrinsic catalytic activity of nanoenzymes introduces the risk of unintended ROS generation outside the tumour environment, particularly if biodistribution is not tightly controlled. While most preclinical studies report minimal acute toxicity, long-term and cumulative toxicity studies remain limited. The degradation behaviour, metabolic fate, and clearance pathways of nanoenzymes vary widely depending on composition, size, surface chemistry, and coating strategy. Without standardised toxicological evaluation frameworks, it remains difficult to directly compare safety profiles across different CAT-PDT systems. From clinical translation perspective, nanoenzymes must demonstrate predictable degradation, efficient clearance, and negligible long-term catalytic activity in healthy tissues [126,129].

Effective CAT-PDT relies on precise co-localisation of nanoenzymes and PS molecules within tumour tissue. However, heterogeneous tumour vasculature, elevated interstitial fluid pressure, and variable permeability significantly influence nanoparticle accumulation. Passive targeting through the enhanced permeability and retention EPR) effect remains inconsistent across tumour types and patient populations, limiting reliable therapeutic delivery. Furthermore, catalytic activity is highly dependent on local substrate availability, pH, oxygen tension, and redox balance. These parameters vary both spatially and temporally within human tumours, making it difficult to predict catalytic performance in patients compared to controlled animal models. Achieving consistent tumour selectivity while minimising off-target accumulation is therefore a critical translational bottleneck. Strategies such as ligand-mediated targeting, biomimetic coatings, stimuli-responsive activation, and cell-mediated delivery systems show promise but add additional complexity to system design and regulatory approval [126,129]. At present, CAT-PDT nanoenzyme platforms remain predominantly at the preclinical stage, with therapeutic efficacy demonstrated mainly in cellular and animal models rather than established clinical studies.

### 5.6. Outlook for Clinical Transition

Despite these challenges, the translational outlook for CAT-PDT remains promising. Advances in materials engineering, bio responsive design, imaging-guided therapy, and computational optimisation are steadily improving the controllability, safety, and predictability of nanoenzyme-based systems. Simplified, degradable, and substrate-efficient nanoenzymes are likely to play a pivotal role in early clinical translation. Moreover, integration with existing clinical PDT infrastructures and established PS molecules may accelerate regulatory progress. Ultimately, successful clinical translation of CAT-PDT will depend on balancing catalytic sophistication with practical feasibility. Systems that prioritize safety, regulatory compliance, and manufacturing simplicity, while retaining sufficient catalytic functionality to overcome TME barriers, are most likely to progress form experimental innovation to clinically meaningful cancer therapies [126,127,129].

## 6. Future Directions

While CAT-PDT demonstrates clear mechanistic advantages over conventional PDT, its translation toward clinical application remains dependent on addressing several unresolved challenges. A comparative overview of conventional PDT and CAT-PDT, highlighting current performance gaps, translational considerations, and levels of clinical maturity, is summarised in Table 4. These distinctions provide a conceptual framework for identifying key research priorities required to advance CAT-PDT toward clinical implementation.

**Table 4:** Comparative overview of conventional photodynamic therapy and catalytic photodynamic therapy.

Feature	Conventional PDT	CAT-PDT
PS delivery	Free, molecular, or carrier-loaded PS with limited tumour accumulation	PS integrated within or co-localised with catalytic nanoenzymes or hybrid systems
Oxygen dependence	Strongly oxygen-dependent	Reduced oxygen dependence due to catalytic oxygen generation
ROS generation profile	Transient, light-limited ROS production	Sustained ROS generation through combined photochemical and catalytic pathways
Performance under hypoxia	Poor	Effective even in hypoxic tumour regions
Sensitivity to antioxidant defences	High	Reduced due to catalytic antioxidant depletion and redox modulation
TME modulation	Minimal	Active modulation (oxygenation, redox disruption, sustained ROS flux)
Mechanistic complexity	Low	High
Key translational challenges	Light penetration, oxygen limitation	Catalytic control, safety, manufacturing scalability, regulatory complexity
Clinical approval	Clinically approved for selected indications	Preclinical
Therapeutic potentials	Effective mainly for superficial, well-oxygenated tumours	High potential for deep, hypoxic, and heterogeneous tumours

Addressing the current limitations of CAT-PDT will require innovation across nanotechnology, imaging, immunology, and conceptual design. The next phase of CAT-PDT and nanoenzyme research is defined by the need to transition from conceptually powerful catalytic systems toward clinically viable therapeutic platforms. One major direction involves the development of substrate-independent nanoenzymes, which can maintain catalytic activity without relying heavily on endogenous H_2_O_2_ or extreme pH. Emerging strategies include pre-loaded catalytic fuels, artificial cofactor systems, and engineered catalytic scaffolds that operate efficiently under physiological conditions. Such approaches could eliminate one of the central bottlenecks highlighted in current TME-dependent catalytic designs [130,131].

Another key opportunity lies in dynamic and programmable nanoenzymes capable of responding to tumour cues in controlled, synchronised manner. Systems that shift catalytic in real time, such as switching between catalase-like and peroxidase-like activity depending on local oxygen tension, could optimise PDT activation across heterogeneous tumour regions. In parallel, enzyme logic-gates and built-in catalytic off-switches are being explored to improve safety by preventing uncontrolled ROS cascades [88].

Extending CAT-PDT to deep-seated tumours remains a significant challenge. Integrating nanoenzymes with self-illuminating modalities such as bioluminescence resonance energy transfer, chemiluminescence or X-ray activated systems may bypass the limitations of external light delivery. This would expand CAT-PDT to deep-seated tumours that are currently inaccessible to optical activation while still preserving catalytic amplification [132].

There is also a growing interest in biohybrid and cell-mediated delivery. Carrier systems such as macrophage hitchhiking, exosomes, and engineered bacteria offer improved tumour penetration and catalytic site specificity. These living or semi-living delivery platforms could spatially guide nanoenzymes into hypoxic cores, where catalytic enhancement is most needed [133].

Finally, computational nanoenzyme design, including machine learning, guided catalyst optimisation, molecular simulation of catalytic pathways, and AI-driven TME modelling, is expected to accelerate the rational design of next-generation CAT-PDT systems, as predictive tools may allow researchers to tune catalytic turnover, substrate affinity, and ROS yields with far greater accuracy than current empirical screening approaches [134].

## 7. Conclusions

CAT-PDT follows the same mechanisms of conventional PDT but extends them by incorporating enzyme-mimetic nanomaterials that overcome limitations related to the TME. Through oxygen generation, ROS production, and modulation of the tumour redox balance, CAT-PDT improves treatment efficacy under conditions that normally limit conventional PDT, including hypoxia, elevated antioxidant activity, and heterogeneous tumour architecture. These catalytic functions facilitate more sustained ROS generation and improve PDT activity throughout the tumour.

Studies across catalase-like, peroxidase-like, oxidase-like, SOD-like, cascade-like catalytic hybrids, and metal–organic catalysts demonstrate that catalytic amplification enhances ROS production and supports more effective PDT. Advances in nanoenzyme design have enabled oxygen generation, substrate cycling, and dual catalytic activity and tumour-responsive behaviour, highlighting the versatility of these materials for improving PDT.

Despite these advances, several challenges must be addressed before CAT-PDT can be translated into clinical practice. Tumour heterogenicity continuous to influence catalytic performance, while uncertainties surrounding long-term nanoenzyme retention, biodegradation, and safety require further investigation. Drug delivery barriers, limited light penetration, and regulatory requirements also remain significant obstacles. Addressing these challenges will be essential for improving reproducibility, safety, and clinical applicability.

Future research is likely to focus on substrate-independent nanoenzymes, self-illuminating activation systems, deep-tissue catalysis, and biohybrid delivery systems. Integrating CAT-PDT with immunotherapy, radiotherapy, or targeted chemotherapy may further improve therapeutic outcomes. Continued advances in nanoenzyme engineering, computational design, and molecular imaging are expected to support the development of safer and more effective catalytic systems for clinical applications.

Overall nanoenzyme-induced CAT-PDT represents a promising approach for improving PDT for cancer therapy by overcoming many of the biochemical limitations of the TME. Continued progress in catalyst engineering, translational science, and clinical validation will determine how rapidly this promising technology moves from experimental innovation to practical oncology.

## Figures and Tables

**Table 1:** ROS-induced biological outcomes in PDT.

ROS Type	Cellular Target	Outcome	Functional Role
Singlet oxygen (^1^O_2_)	Lipids, proteins, membranes	Apoptosis, necrosis	Primary cytotoxic mediator in classical PDT; short-lived; highly reactive
Hydroxyl radicals (•OH)	DNA, proteins, membranes	Necrosis	Extremely reactive; acts locally; generated mainly through Type I pathway
Superoxide (O_2_•^−^)	Mitochondria, ER	Oxidative stress; apoptosis	Can dismantle to H_2_O_2_; contributes to oxidative signalling
Hydrogen peroxide (H_2_O_2_)	Cytoplasm, nucleus	Apoptosis, oxidative damage	Longer-lived ROS; can diffuse farther and amplify cellular damage

**Table 3:** Classification and catalytic functions of nanoenzymes for CAT-PDT.

Nanoenzyme Class	Natural Enzyme Mimicked	Primary Catalytic Reaction	CAT-PDT Mechanism	Typical Nanoenzymes
Catalase-like	Catalase	2H_2_O_2_ → 2H_2_O + O_2_	Increases intratumoral O_2_ supply; relieves hypoxia; enhances Type II PDT	MnO_2_ nanoparticles, MOFs
Peroxidase-like	Peroxidase	H_2_O_2_ + substrate → •OH or oxidised products	Produces strong radicals; oxygen-independent ROS pathway; complements light-triggered ROS	Fe_3_O_4_, CuO, and Co_3_O_4_
Oxidase-like	Oxidases	Substrate + O_2_ → Product + H_2_O_2_	Produces H_2_O_2_ from tumour metabolites; provides continuous ROS substrate supply; primes cells for enhanced PDT	Pt nanoparticles, MOFs, Au nanoparticles, carbon-based nanoenzymes
Superoxide dismutase (SOD)-like	SOD	2 O_2_•^−^ + 2H^+^ → H_2_O_2_ + O_2_	Generates additional H_2_O_2_; replenishes substrate for catalase, peroxidase; increases ROS cycling	CeO_2_, Mn nanoparticles
Metal–organic framework (MOF) nanoenzymes	Multiple	Metal node + ligand → modular catalytic transformations	Highly tuneable; can combine PDT and catalytic sites in one system; enhances ROS diffusion and PS loading; supports multi-enzyme catalysis	
Multi-enzyme nanoenzymes	Two or more enzymes combined	Sequential multi-step catalysis	Enhances ROS through cascade amplification; integrates oxygen-producing and oxygen-independent pathways; efficient in severely hypoxic tumours	CeO_2_-MnO_2_ hybrids; Fe-Cu bimetallics

## Data Availability

No new data were created or analyzed in this study. Data sharing is not applicable to this article.

## Abbreviations

CAT-PDT	Catalytic photodynamic therapy
DAMPs	Danger-associated molecular patterns
ECM	Extracellular matrix
GSH	Glutathione
HIFs	Hypoxia-inducible factors
ICD	Immunogenic cell death
MOF	Metal–organic framework
PS	Photosensitiser
PDT	Photodynamic therapy
ROS	Reactive oxygen species
SOD	Superoxide dismutase
TME	Tumour microenvironment
